# Efficacy of Dry Needling and Acupuncture in Patients with Fibromyalgia: A Systematic Review and Meta-Analysis

**DOI:** 10.3390/ijerph19169904

**Published:** 2022-08-11

**Authors:** Juan Antonio Valera-Calero, César Fernández-de-las-Peñas, Marcos José Navarro-Santana, Gustavo Plaza-Manzano

**Affiliations:** 1 VALTRADOFI Research Group, Department of Physiotherapy, Faculty of Health, Universidad Camilo José Cela, 28692 Villanueva de la Cañada, Spain; 2 Department of Physical Therapy, Occupational Therapy, Rehabilitation and Physical Medicine, Universidad Rey Juan Carlos, 28933 Alcorcón, Spain; 3 Cátedra Institucional en Docencia, Clínica e Investigación en Fisioterapia: Terapia Manual, Punción Seca y Ejercicio Terapéutico, Universidad Rey Juan Carlos, 28933 Alcorcón, Spain; 4 Faculty of Health, Universidad Católica de Ávila (UCAV), 05005 Ávila, Spain; 5 Grupo InPhysio, Instituto de Investigación Sanitaria del Hospital Clínico San Carlos, 28040 Madrid, Spain; 6 Department of Radiology, Rehabilitation and Physiotherapy, Universidad Complutense de Madrid, 28040 Madrid, Spain

**Keywords:** fibromyalgia, dry needling, acupuncture, chronic diseases, systematic review

## Abstract

Fibromyalgia (FM) is a syndrome that involves chronic pain, fatigue, sleep disturbance and impaired quality of life and daily functioning. In addition to medical and psychological therapies, other therapies including acupuncture and dry needling aim to reduce pain and disability in patients with FM. The aim of this study was to investigate the efficacy of dry needling and acupuncture in patients with FM regarding pain, function and disability in both the short and the long term. MEDLINE, PubMed, SCOPUS and Web of Science databases were systematically searched for randomized controlled trial studies evaluating efficacy data of dry needling or/and acupuncture treatments to improve pain, fatigue, sleep disturbance and impaired quality of life and/or daily function. A qualitative analysis including the methodological quality and a systematic data synthesis was performed. A total of 25 studies addressed the selection criteria. Most studies had an acceptable methodological quality. Four studies assessed the effect of dry needling, and twenty-one studies assessed the effect of acupuncture. In general, both interventions improved pain, anxiety, depression, fatigue, stiffness, quality of sleep and quality of life. However, both techniques were not compared in any study. Acupuncture and dry needling therapies seems to be effective in patients with FM, since both reduced pain pressure thresholds, anxiety, depression, fatigue, sleep disturbances and disability in the short term. It is still required to compare both techniques and their application in the long term.

## 1. Introduction

Fibromyalgia (FM) is a condition that involves generalized chronic pain [1] associated with fatigue, sleep disturbance, depression and cognitive impairments [2,3]. This disease can affect people at different ages, but FM is most frequently found in middle-aged women [4]. In addition to gender and age, prior family history of FM increases the risk of suffering this condition. Therefore, this suggests a mixed genetic and lifestyle etiology [5], but the exact etiology is still unknown. Previous studies assessed the altered pain perception reporting a chronic and increased pain response to a painful stimulus (hyperalgesia) and pain caused by a stimulus which normally should not cause pain (allodynia) [6,7,8]. Gender differences were found in the intensity, frequency, duration and locations, female patients being more affected than male patients. Although pain perception is conditioned by subjective and personal experiences, these differences between genders could be explained by biologic features related to endogenous pain-relief mechanisms or the influence of gonadal hormones [9].

Some common treatments used for patients with FM to reduce fatigue, sleep disturbance and psychological and physical symptoms are psychological treatments (e.g., cognitive behavioral therapy) [10], aerobic exercise [10], and pharmacological treatment (e.g., amitriptyline, anticonvulsants, noradrenaline and serotonin reuptake inhibitors) [11]. However, these pharmacological options were demonstrated to be not effective at all [12] and to present adverse effects that make their use difficult in the long term, and a multidimensional approach including patient education, behavioral therapy, exercise and pain management is recommended [13]. Dry needling and acupuncture are complementary treatment options in this multidimensional approach that could be potentially used in the long term for reducing pain and disability in this population that have been widely studied in several conditions. 

Dry needling is a therapeutic procedure that consists of a needle insertion with any pharmacological substance into a myofascial trigger point (MTrP) to inactivate it and reduce the pain [14]. This mechanism has been widely studied in several conditions (e.g., acute and chronic low back pain, myofascial pain syndrome and whiplash) [15] and showed a similar response to lidocaine injections in the treatment of MTrP symptoms (e.g., pain relief, increased range of movement and improved quality of life) [16]. On the other hand, acupuncture is a complementary and alternative medicine treatment that has been used to treat different conditions, including chronic pain, for over three millennia in China [17] and is used by up to 91% of the patients with FM [18]. Both techniques could base their analgesic effect on the neurotransmitter and hormone regulation on the central nervous system by stimulating nerve fibers (e.g., A delta afferences) and producing an activation in the cascade of pain-modulating endorphins, serotonin and noradrenaline, which contributes to analgesia [19].

Although dry needling and acupuncture are applied to decrease pain in different musculoskeletal conditions and several systematic reviews assessed the efficacy of acupuncture in patients with FM during the last 10 years [13,18,20,21], the inclusion of dry needling assessment for the management of pain, function and disability in patients with FM was not considered in any prior systematic review. Therefore, the current systematic review with meta-analysis evaluates the efficacy of both dry needling and acupuncture to improve pain, function and disability in the FM population.

## 2. Materials and Methods

### 2.1. Study Design

This systematic review and meta-analysis followed the Preferred Reporting Items for Systematic Reviews and Meta-Analyses (PRISMA) statement [22]. The international OSF Registry registration link for this study is https://doi.org/10.17605/OSF.IO/Y4627 (registered on 25 October 2020).

### 2.2. Data Sources

Electronic literature searches were conducted on MEDLINE, PubMed, SCOPUS and Web of Science databases from their inception to 22 May 2022. Bibliographical search strategies were conducted with the assistance of an experienced health science librarian and FM management following the guidelines described by Greenhalgh [23]. Search strategies were based on a combination of Mesh terms and keywords following the PICO (Population, Intervention, Comparison, Outcome) question:

Population: Adults (older than 18 years old) with FM;

Intervention: Use of dry needling and/or acupuncture;

Comparator: Active control interventions;

Outcomes: Pain (e.g., subjective pain perception, pressure pain thresholds and/or pain questionnaires), Severity of the condition (e.g., fibromyalgia, anxiety, fatigue and/or depression severity indexes), and/or quality of life (e.g., quality of life, sleep disturbances and/or health questionnaires).

An example of the search strategy (PubMed database) is as follows Box 1:

Box 1 **Example of the search strategy (PubMed database).**

Filters: [Title/Abstract]

#1 Fibromyalgia [MeSH]; #2 Fatigue Syndrome, Chronic [MeSH]; #3 Tender Points
 
#4 #1 OR #2 OR #3
 
#5 Needling, Dry [MeSH]; #6 Acupuncture [MeSH]; #7 Acupuncture Therapy [MeSH]; #8 Acupuncture Analgesia [MeSH]
 
#9 #5 OR #6 OR #7 OR #8
 
#10 Pain [MeSH]; #11 Chronic Pain [MesH]; #12 Pain Perception [MeSH]; #13 Pain Threshold [MeSH]; #14 Anxiety [MeSH]; #15 Depressive Disorder [MeSH]; #16 Fatigue [MeSH]; #17 Quality of Life [MeSH]; #18 Sleep [MeSH]; #19 Sleep Disorders
 
#20 #10 OR #11 OR #12 OR #13 OR #14 OR #15 OR #16 OR #17 OR #18 OR #19

### 2.3. Study Eligibility Criteria

Studies were eligible for inclusion if they evaluated the application of dry needling and/or acupuncture in adult patients with FM for the pain, disability, function and/or quality of life management and were published during the last 10 years in the English, Portuguese or Spanish languages. Animal studies, cadaveric studies, published proceedings, abstracts and studies with a sample size lower than 30 subjects were excluded for review. All included studies must have obtained approval from an ethics committee or institutional review board.

### 2.4. Study Appraisal and Synthesis Methods

The Mendeley Desktop v.1.19.4 for Mac OS (Glyph & Cog, LLC 2008) program was used to insert the search hits from the databases. First, the duplicates were removed. Second, title/abstracts of the articles were screened for potential eligibility by two authors. Third, the full text was analyzed to identify potentially eligible studies. Reviewers were required to agree in the inclusion/exclusion decision. In case of discrepancy between the initial two reviewers, a third reviewer participated in the process to reach the consensus for including the study in the systematic review or not.

A standardized data extraction form containing questions on study design, sample size, objectives, interventions, outcomes assessed, results and conclusions was used, following the main structure reported by Shokraneh et al. [24]. The methodological quality of the included studies was assessed using the PEDro scale, which consists of a checklist of 10 scored yes-or-no questions pertaining to the internal validity and the statistical information provided. Reported cut-offs for the scale interpretation are as follows: 0–3 score was interpreted as poor quality; 4–5 as fair quality; and 6–10 as high quality [25].

### 2.5. Level of Evidence

The Grading of Recommendations Assessment, Development and Evaluation (GRADE) approach was used to evaluate the level of evidence [26]. The level of evidence was classified as high, moderate, low or very low based on study limitations, indirectness of evidence, unexplained heterogeneity, imprecision of the results and high probability of publication bias [27]. High-quality evidence was scored when all items were negative; moderate quality was scored when one item included serious risk, low quality was scored if two items showed serious risk or one item showed very serious risk; or very low quality was scored when three or more items had serious risk or two or more had very serious risk. This process was also performed by two authors, with the participation of a third one if disagreement occurred.

### 2.6. Data Analysis

Data analysis was performed with Review Manager statistical software (RevMan version 5.3). Data synthesis was presented by groups according to comparative groups, such as sham/control/placebo; manual therapy or other physical therapy intervention; and by follow-up, such as short- (0 to 3 months) and mid-term (>3months to <6 months), since long-term (≥6 months) data were not available. No other subgroup analysis was prespecified a priori.

Data extraction for the data analysis included sample size, means and standard deviations of the outcomes. When the trial reported standard errors, they were converted to standard deviations. Mean and standard deviations were estimated from graphs when needed. If data were expressed as median and interquartile range, they were converted to mean and standard deviation as needed [28]. 

The between-groups mean difference (MD) with the 95% confidence interval (CI) was calculated for those outcomes assessed with the same instrument, e.g., pain intensity and pressure pain thresholds. Between-groups mean differences were converted to SMD when different instruments were used for the same outcome, e.g., pain-intensity. A random-effects model was used to determine the effect sizes (SMD). An effect size (SMD) of ≥0.8 was considered large, between 0.5 and 0.8 was considered moderate and between 0.2 and 0.5 was considered small [29]. *p*-values < 0.05 were considered statistically significant.

Finally, when two subgroups included the same intervention, e.g., dry needling, the sample size was adjusted by dividing the sample size as the Cochrane textbook recommends for avoiding duplication in the overall effect [30]. 

The I^2^ statistic was applied to determine the heterogeneity between the included trials. We used the following interpretation: 0–40% represented no relevant heterogeneity; 30–60% represented moderate heterogeneity; 50–90% suggested substantial heterogeneity; and 75–100% suggested considerable heterogeneity [31]. 

## 3. Results

### 3.1. Study Selection

**Figure 1:**
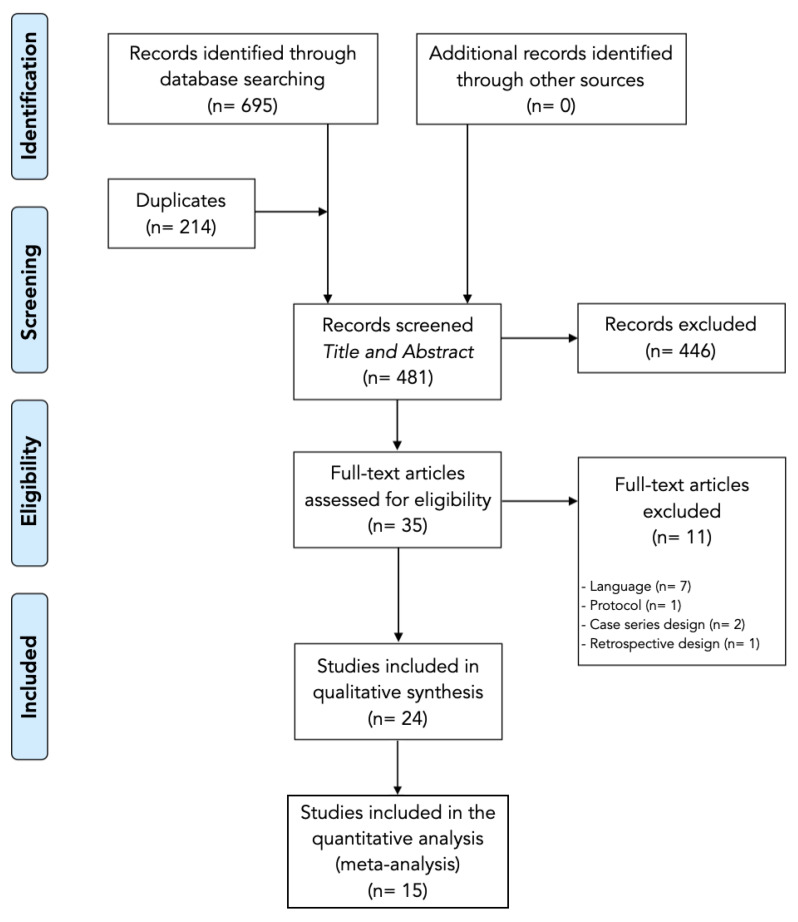
Preferred Reporting Items for Systematic Reviews and Meta-Analyses Flowchart.

The electronic searches identified 695 potential studies for review. After removing 214 duplicated studies, 481 studies remained. Four hundred forty-six (*n* = 446) studies were excluded based on examination of their titles or abstracts, leaving 35 articles for full-text analysis. Eleven articles were excluded because they were not published in English or Spanish languages (*n* = 8), were protocols (*n* = 1) or had a case series design (*n* = 2) or retrospective observational design (*n* = 1). A total of twenty-four studies were included in this systematic review [6,32,33,34,35,36,37,38,39,40,41,42,43,44,45,46,47,48,49,50,51,52,53,54]. The included articles investigated dry needling (*n* = 4) or acupuncture (*n* = 20), but none compared both techniques directly in the same study (Figure 1).

### 3.2. Methodological Quality Assessment

The methodological quality scores measured using the PEDro scale ranged from 5 to 9 (mean: 6.8; SD: 1.1) out of a maximum of 10 points (Table 1). The most consistent flaws were the blinding of all therapists administering the therapy (*n* = 22 studies out of 25), the blinding of the subjects to discriminate if they had or did not have the treatment (*n* = 19 out of 25), the concealed allocation (*n* = 10 out of 25) and the blinding of all assessors who measured at least one key outcome (*n* = 10 out of 25). 

Overall, most of the studies specified the eligibility criteria (*n* = 25), allocated the subjects randomly to the groups (*n* = 23), had balanced group at baseline regarding the most important prognostic indicators (*n* = 21), obtained data from more than 85% of the subjects initially allocated in the groups (*n* = 20), participants received the treatment or control condition as allocated (*n* = 18), statistical comparisons between groups were reported (*n* = 25) and both point measures and measures of variability provided (*n* = 25). The methodological quality of studies assessing dry needling was higher (mean: 7.2; SD: 1.7) than that of acupuncture (mean: 6.6; SD: 1.0).

### 3.3. Dry Needling

Table 2 summarizes the four studies investigating the efficacy of dry needling in patients with FM. Just one study applied the technique in Tender Points (TP) [36] while three applied dry needling in MTrPs [6,42,50]. Comparative interventions selected for these studies were their ordinary medical treatment [30], taping applications [42], manual myofascial release techniques [6], and Transcutaneous Electrical Nerve Stimulation (TENS) [50]. The included studies had a total sample size of 312 patients with FM (35 men and 277 women).

Although pain was a common outcome in all the included studies [6,36,42,50], the measurement instruments were not consistent in all the designs. Visual Analogue Scale (VAS) of pain was used in two studies [36,50], Pain Pressure Thresholds (PPTs) were reported in three of the studies for both TPs [36] and MTrPs [6,42] and McGill Pain Questionnaire and myalgic score in just one study [30]. In addition to pain outcomes, quality of life was assessed in two studies, including questionnaires, sleep quality, impact of fibromyalgia and fatigue [6,36]; psychological outcomes including depression and anxiety were assessed in one study [6]; spinal mobility was assessed in one study [42]; and finally, autonomic function including heart rate variability, galvanic skin response, oxygen saturation (SpO_2_) and photoplethysmography was assessed in one study [50].

In general, dry needling is shown to induce improvements in short-term subjective pain perception, pain pressure thresholds, mobility, fatigue, quality of life, physical function, physical role, general health, vitality, social function, emotional role and mental health. 

### 3.4. Acupuncture 

Twenty studies investigated the effects of acupuncture in patients with FM (Table 3). In the final qualitative analysis, four quasi-experimental studies [32,34,43,51], one experimental effectiveness comparative study [49] and sixteen controlled trials (of which fifteen were randomized [33,35,37,38,39,41,44,45,46,47,48,52,53,54], one non-randomized [40] and two double-blinded [38,41]) were included.

This study selection compared acupuncture with dietary therapy with and without acupuncture [33,53], somatic and abdominal acupuncture [34], with scalp acupuncture [35,37], with moxibustion [37,40], with music and/or vibratory therapy [39], electroacupuncture [40], simulated acupuncture [46], patients’ education [47], TENS [48,49], core stability training [46] and physiotherapy [54]. However, the most selected comparator group was the application of sham acupuncture [37,38,41,44,45,46]. Most of the total participants (*n* = 1497) were women (*n* = 1481). Just two studies included a male sample (*n* = 16) [38,51].

The acupuncture points most commonly treated were PC-6 (Neiguan), LI-4 (Hegu), GB-34 (Yanglingquan), SP-6 (Sanyinjiao), Du-14 (Da Zhui), Si- 3 (Houxi), Si-4 (Wangu), Si-15 (Jian Zhong Shu), Li-4 (He Gu), Li-11 (Qu Chi), H-7 (Shen Men), P-6 (Nei Guan), Ren-6 (Qihai), Liv-3 (Tai Chong), St-36 (Zu San Li), LR-3 (Taichong), UB-11 (Tianzhu), UB-12 (Fengmen), UB-13 (Feishu), UB-14 (Jueyinshu), GB-21 (Jianjing), UB-62 (Shenmai), UB-64 (Jinggu), UB-65 (Shugu) and Sp-6 (San Yin Jiao) [32,33,34,35,37,38,39,40,41,43,44,45,46,47,48,49,51,54].

The most assessed outcomes were the effect of acupuncture on pain (seventeen studies) measured by using a VAS [32,33,34,35,37,38,39,41,43,44,45,46,48,49,54], the McGill Questionnaire [49], PPTs [40,48], the Pain Catastrophizing Scale [51], the Pain Detect Questionnaire [51] or the Wong-Baker Faces Pain Scale [34], and the effect on fibromyalgia severity (seventeen studies) was assessed by the Fibromyalgia Impact Questionnaire (FIQ) or the number of tender points [32,33,34,35,37,39,41,42,43,44,46,47,48,49,51,52,53,54]. Thus, psychological features (e.g., anxiety and depression) were assessed in seven studies [34,41,44,46,48,54], biomarkers in one study [43], balance in one study [52] and fatigue in four studies [44,47,52,53].

In general, acupuncture and scalp acupuncture showed to be effective in the treatment of fibromyalgia for reducing pain and disability in the short term if combined with other treatments, reducing the adverse effects and treatment costs. Although the placebo effect seems to play a relevant role in these improvements since sham acupuncture demonstrated significant reduction in pain and disability, real treatment seems to be more effective.

### 3.5. Meta-Analysis Results

#### 3.5.1. Pain Intensity

**Figure 2:**
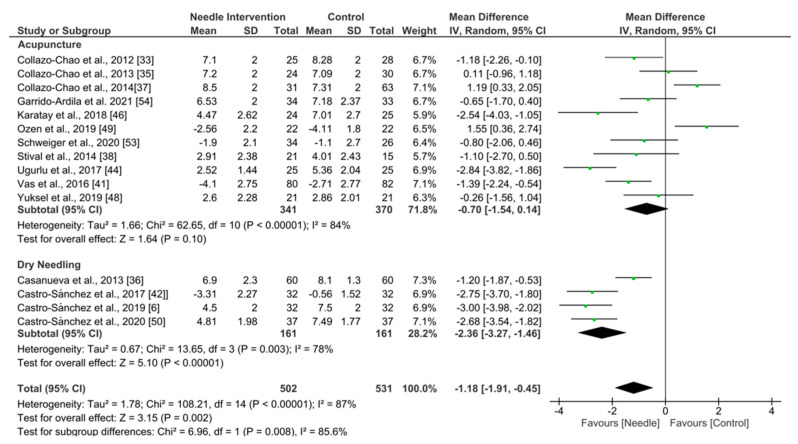
Forest plot for Pain Intensity after 0–3 months.

Needle interventions exhibited an overall significant effect (MD −1.18, 95% CI −1.91 to −0.45; *p* = 0.002 Z = 3.15; SMD −0.55, 95% CI −0.89 to −0.21, *N* = 1033, *n* = 15 trials) for reducing pain after the intervention to 3 months follow-up vs. a comparison group but with substantial heterogeneity (I^2^ = 85%) between the trials (Figure 2). A significant effect was found in the dry needling subgroup (MD −2.36, 95% CI −3.27 to −1.46, *p* = 0.003, I^2^ = 78%; SMD −1.20, 95% CI −1.66 to −0.75, *N* = 322, *n* = 4 trials) but not for acupuncture (MD −0.70, 95% CI −1.54 to −0.14, *p* = 0.10, I^2^ = 84%; SMD −0.30, 95% CI −0.65 to −0.06, *N* = 711, *n* = 11 trials). Testing for subgroup differences showed significant differences between groups (*p* = 0.0008, I^2^ = 85.6%). The funnel plot did not present potential publication bias.

**Figure 3:**
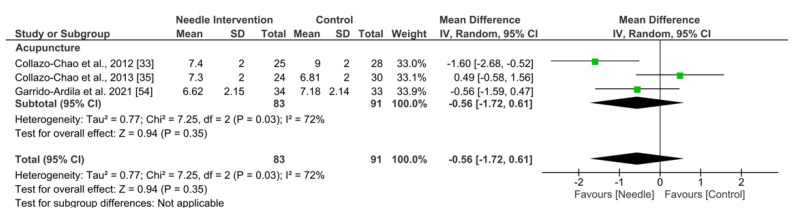
Forest plot for Pain Intensity: 3 to 6 months.

Acupuncture exhibited an overall nonsignificant effect (MD −0.56, 95% CI −1.72 to 0.61; *p* = 0.35 Z = 0.94; SMD −0.26, 95% CI −0.82 to 0.29, *N* = 174, *n* = 3 trials) for reducing pain after 3 to 6 months of follow-up vs. a comparison group but with substantial heterogeneity (I^2^ = 70%) between the trials (Figure 3). 

**Figure 4:**
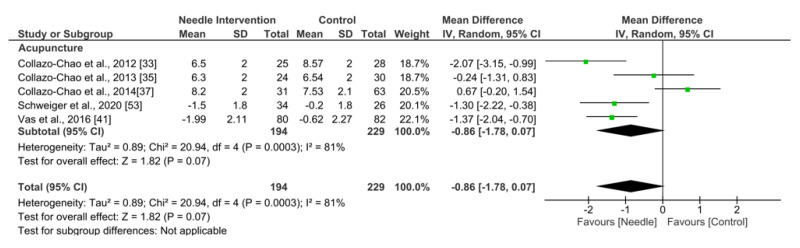
Forest plot for Pain Intensity: +6 months.

Acupuncture exhibited an overall nonsignificant effect (MD −0.86, 95% CI −1.78 to 0.07; *p* = 0.07 Z = 1.82; SMD −0.42, 95% CI −0.88 to 0.04, *N* = 423, *n* = 35trials) for reducing pain after 6 months follow-up or more vs. a comparison group but with substantial heterogeneity (I^2^ = 81%) between the trials (Figure 4). 

#### 3.5.2. Fibromyalgia Impact Questionnaire

**Figure 5:**
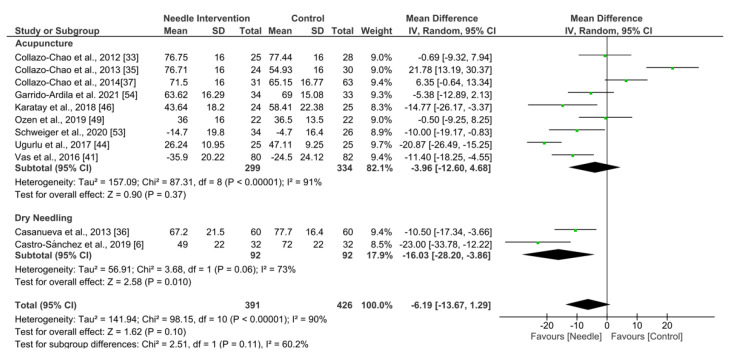
Forest plot for FIQ after 0–3 months.

Needle interventions exhibited an overall nonsignificant effect (MD −6.19, 95% CI −13.67 to 1.29; *p* = 0.10 Z = 1.62; SMD −0.36, 95% CI −0.77 to 0.05, *N* = 817, *n* = 11 trials) for FIQ after the intervention to 3 months follow-up vs. a comparison group but with substantial heterogeneity (I^2^ = 87%) between the trials (Figure 5). A significant effect was found in the dry needling subgroup (MD −16.03, 95% CI −28.20 to −3.86, *p* = 0.010, I^2^ = 73%; SMD −0.75, 95% CI −1.22 to −0.28, *N* = 184, *n* = 2 trials) but not for acupuncture (MD −0.70, 95% CI −1.54 to −0.14, *p* = 0.10, I^2^ = 84%; SMD −0.26, 95% CI −0.75 to 0.22, *N* = 633, *n* = 9 trials). Testing for subgroup differences did not show significant differences between groups (*p* = 0.11, I^2^ = 60.2%). The funnel plot did not present potential publication bias.

**Figure 6:**
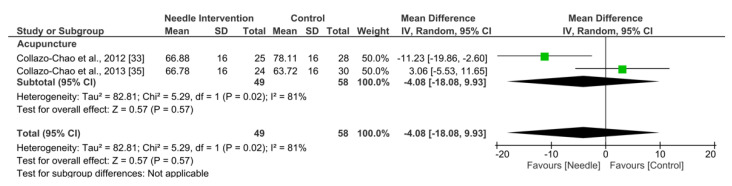
Forest plot for FIQ: 3–6 months.

Acupuncture exhibited an overall nonsignificant effect (MD −4.08, 95% CI −18.08 to 9.93; *p* = 0.57 Z = 0.57; SMD −0.25, 95% CI −1.11 to 0.61, *N* = 107, *n* = 2 trials) for FIQ after 3 to 6 months follow-up vs. a comparison group but with substantial heterogeneity (I^2^ = 80%) between the trials (Figure 6). 

**Figure 7:**
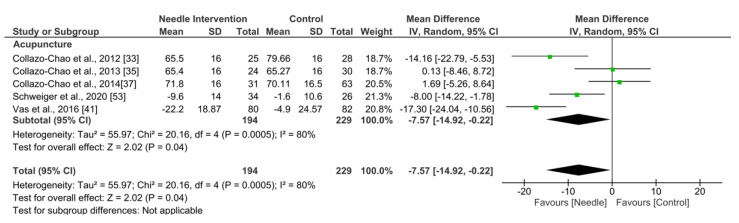
Forest plot for FIQ after +6 months.

Acupuncture exhibited an overall nonsignificant effect (MD −7.57, 95% CI −14.92 to −0.22; *p* = 0.04 Z = 2.02; SMD −0.44, 95% CI −0.85 to −0.02, *N* = 423, *n* = 5 trials) for FIQ after 6 months or more follow-up vs. a comparison group but with substantial heterogeneity (I^2^ = 75%) between the trials (Figure 7). 

#### 3.5.3. Sleeping/Resting Quality

**Figure 8:**
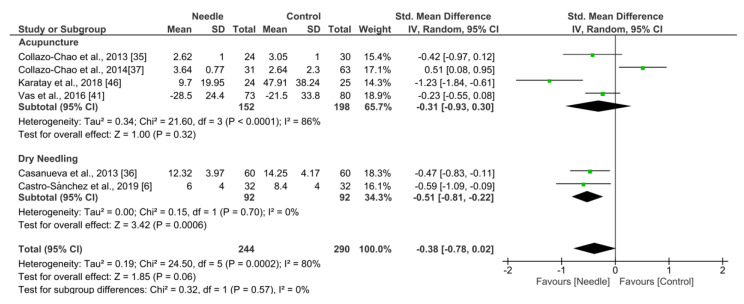
Forest plot for Sleeping Quality after up to 3 months.

Needle interventions exhibited an overall nonsignificant effect (SMD −0.38, 95% CI −0.78 to 0.02, *N* = 534, *n* = 6 trials) for sleep or rest after the intervention to 3 months follow-up vs. a comparison group but with substantial heterogeneity (I^2^ = 80%) between the trials (Figure 8). A significant effect was found in the dry needling subgroup (SMD −0.51, 95% CI −0.81 to −0.22, *p* = 0.0006, *N* = 184, *n* = 2 trials) but not for acupuncture (SMD −0.31, 95% CI −0.93 to 0.30, *p* = 0.32, I^2^ = 86%, *N* = 350, *n* = 4 trials). Testing for subgroup differences did not show significant differences between groups (*p* = 0.57, I^2^ = 0%). 

**Figure 9:**
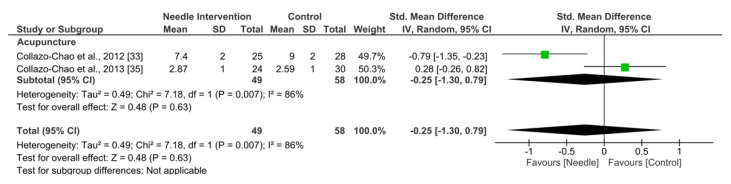
Forest plot for Sleeping Quality after 3–6 months.

Acupuncture exhibited an overall nonsignificant effect (SMD −0.25, 95% CI −1.30 to 0.79, *N* = 107, *n* = 2 trials) for improving sleep after 3 to 6 months follow-up vs. a comparison group but with substantial heterogeneity (I^2^ = 86%) between the trials (Figure 9). 

**Figure 10:**
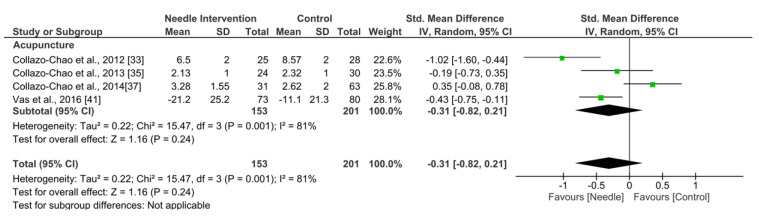
Forest plot for Sleeping Quality after +6 months.

Acupuncture exhibited an overall nonsignificant effect (SMD −0.31, 95% CI −0.82 to 0.21, *N* = 423, *n* = 4 trials) for improving sleep after 6 months or more follow-up vs. a comparison group but with substantial heterogeneity (I^2^ = 81%) between the trials (Figure 10). 

#### 3.5.4. Depression

**Figure 11:**
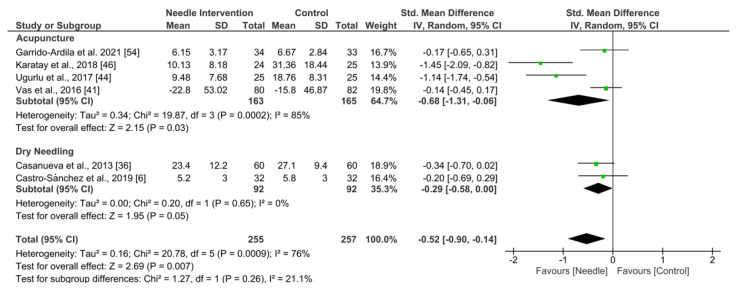
Forest plot for Depression.

As shown in Figure 8, needle interventions exhibited an overall nonsignificant effect (SMD −0.52, 95% CI −0.90 to −0.14, *p* = 0.007, *N* = 512, *n* = 6 trials) for depression after the intervention to 3 months follow-up vs. a comparison group but with substantial heterogeneity (I^2^ = 76%) between the trials (Figure 11). A significant effect was found in the acupuncture subgroup (SMD −0.68, 95% CI −1.31 to −0.06, *N* = 328, *n* = 4 trials) and for dry needling (SMD −0.29, 95% CI −0.58 to 0.00, *N* = 184, *n* = 2 trials). Testing for subgroup differences did not show significant differences between groups (*p* = 0.26, I^2^ = 21.1%).

#### 3.5.5. Pressure Pain Threshold

**Figure 12:**
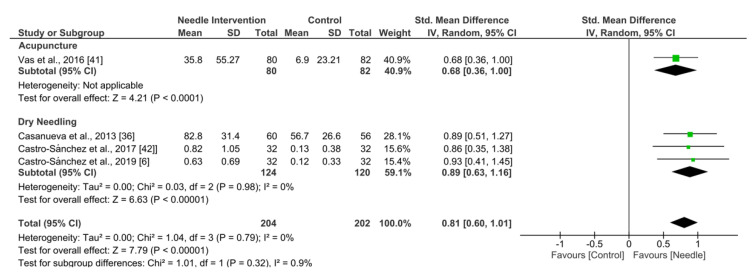
Forest plot for PPTs.

Needle interventions exhibited an overall nonsignificant effect (SMD 0.81, 95% CI −0.60 to 1.01, *p* < 0.00001, *N* = 406, *n* = 4 trials) for PPT after the intervention to 3 months follow-up vs. a comparison group but with substantial heterogeneity (I^2^ = 0%) between the trials (Figure 12). 

### 3.6. Level of Evidence

The risk of bias, inconsistency of the results, indirectness of evidence, imprecision of results and publication bias for determining the level of evidence according to GRADE assessment are detailed in Table 4. The serious/very serious inconsistency of the results (heterogeneity) downgraded the evidence level of dry needling and acupuncture to low or moderate for most of the outcomes assessed, except for the pressure pain threshold in the short-term (with moderate to high quality evidence, especially for dry needling).

## 4. Discussion

The main findings of this meta-analysis were that dry needling could induce a short-term improvements in pain, quality of life, vitality, social function, psychological and severity in patients with FM in comparison with other techniques (e.g., manual myofascial release, cross tape) and that acupuncture, if combined with other treatments, is also an effective technique to reduce the ingestion of analgesics and to improve pain perception, quality of life, pain perception, depression and anxiety from the short-term to up to 12 months. Recent studies highlighted the importance of these outcomes in FM severity [55,56,57,58,59,60,61,62,63]. However, we could not find any study comparing the effectiveness of both techniques within the same research. Although the blinding process in studies including needling techniques is not always possible, the studies included in this review did not consider important methodological aspects regarding allocation concealing, the representative sample size or the inclusion of a proper comparator group, furthering the blinding of therapists, patients and assessors. Therefore, future studies should consider reducing methodological flaws including a greater patient recruitment to correct the patients lost during follow-up, especially in those with large follow-up times, and the inclusion of a blinded assessor to perform the measurements.

Although this is not the first meta-analysis comparing the application of dry needling and acupuncture for reducing pain and disability in patients with FM [64], the existing literature included a minor number of articles (including 14 studies for qualitative synthesis and 8 in the quantitative synthesis) and outcomes (i.e., FIQ, PPT and quality of life) in their analyses compared with this one, despite all types of invasive techniques being compared in their analyses.

One likely reason that could explain the limited number of studies applying dry needling in this population could be that dry needling focuses on MTrPs rather than tender points. MTrPs are defined as hyperirritable spots in skeletal muscles that are associated with hypersensitive palpable and painful nodules in a taut band that can induce referred pain, tenderness, motor dysfunction and autonomic phenomena, probably caused by the ischemia and hypoxia produced by the capillary vessels’ compression in these taut bands, and producing peripheral sensitization [65]. Low blood oxygen levels result in a significative pH reduction, an activation of acid-sensing ion channel receptors, acetylcholinesterase inhibition, ATP stimulation, bradykinin, tumor necrosis factor alfa, interleukins, serotonin, noradrenaline, P substance and calcitonin gen-related peptide [66]. On the other hand, tender points were defined by the American College of Rheumatology assessing the hypersensitivity of 18 specific locations to confirm the clinical diagnosis of FM [67]. 

Based on available literature to date, we found that dry needling induces a global subjective, pain, quality of life and disability improvement in the short-term up to 6 weeks after the treatment [36,50], reporting better results than TENS [50], manual myofascial release treatment [6] and cross tape for all the measured outcomes, except spinal mobility after cross tape treatment [42]. However, further research is needed to confirm these findings due to the lack of studies, the limited comparator groups and the limited follow-up time.

We also found that acupuncture is also an effective and safe tool for managing patients with FM if combined with other therapies (e.g., their usual medication or dietary therapy). Collazo-Chao et al. [32] reported a decrease in the analgesic intake, further improving disability, sleep quality and intensity of pain. All the acupuncture techniques assessed demonstrated similar results (e.g., somatic, abdominal and scalp) and agreed with these positive effects. However, there is enough evidence proposed by Webber et al. [39], Ugurlu et al. [44], Zucker et al. [45] and Karatay [46] to state the substantial role of the placebo effect, even if the verum acupuncture showed better results than sham acupuncture [41]. Further studies could compare the changes in serum serotonin and substance P levels both in the application of dry needling and acupuncture to explain analgesic mechanisms differences depending on the application procedures [46]. These conclusions must be interpreted carefully due to the methodological flaws found in these studies since most of them had no comparator groups or the group assignation was not randomized. 

None of the studies assessed in the meta-analysis conducted by Deare et al. [18] nor Yang et al. [13] were included in this systematic review due to the publication date or the sample size. However, our conclusions are consistent with all the previous systematic reviews [13,18,21] since they reported low-to-moderate-level evidence that, compared with no treatment and standard therapy, acupuncture improves pain and stiffness in people with FM and moderate-level evidence that the effect of acupuncture does not differ from sham acupuncture for reducing FM symptoms. Thus, the methodological quality flaws that we found in this study are similar to those reported in these prior systematic reviews, this being the main reason for the level of evidence weakness.

### Limitations

Finally, there are some limitations of the current systematic review. First, we have only included articles written in the English or Spanish language, so we have discarded some relevant published studies in other languages, such as Chinese, with a high number of potentially relevant acupuncture studies. Furthermore, we did not include those studies which were accepted but unpublished. Secondly, the limited number of studies assessing dry needling in FM populations as well the inclusion of quasi-experimental acupuncture studies with no comparative group in this review are the main reason to advise a careful interpretation of our conclusions. Further research with proper comparators and blinding is needed to reinforce to recommend the use of dry needling or acupuncture in patients with FM. 

## 5. Conclusions

Due to the lack of studies assessing dry needling and the methodological quality flaws of the studies assessing acupuncture, this systematic review should be interpreted carefully. Overall, there is a low-to-moderate-quality level of evidence that suggests that dry needling is effective for improving pain, disability and quality of life in the short term (up to 6 weeks). The same level of evidence supports acupuncture as an effective complementary treatment to medication and exercise for improving FM severity and symptoms including pain, sleep quality, quality of life, depression, anxiety and fatigue. We did not find any research comparing both techniques in the same study. Further research is needed considering the inclusion of a proper comparator group, larger sample sizes and patient, therapist and/or assessor blinding, if possible, to make more consistent recommendations.

## Figures and Tables

**Table 1:** Methodological Quality Assessment using PEDro Scale of the Included Studies.

Study	Item No.											Total
	1	2	3	4	5	6	7	8	9	10	11	0–10
Collazo-Chao et al., 2010 [32]	+	−	−	+	−	−	−	+	+	+	+	5
Collazo-Chao et al., 2012 [33]	+	+	+	−	−	−	+	+	−	+	+	6
Iannuccelli et al., 2012 [34]	+	+	−	+	+	−	−	+	+	+	+	7
Collazo-Chao et al., 2013 [35]	+	+	−	−	−	−	+	+	+	+	+	6
Casanueva et al., 2014 [36]	+	+	+	+	−	−	−	−	−	+	+	5
Collazo-Chao et al., 2014 [37]	+	+	−	−	−	−	+	+	+	+	+	6
Stival et al., 2014 [38]	+	+	−	+	+	−	+	−	−	+	+	6
Weber et al., 2015 [39]	+	+	+	+	−	−	−	+	−	+	+	6
Dias et al., 2016 [40]	+	+	−	+	−	−	−	−	+	+	+	5
Vas et al., 2016 [41]	+	+	+	+	−	−	+	+	+	+	+	8
Castro-Sánchez et al., 2017 [42]	+	+	+	+	−	−	+	+	−	+	+	7
Iannuccelli et al., 2017 [43]	+	+	−	+	−	−	−	+	+	+	+	6
Ugurlu et al., 2017 [44]	+	+	−	+	+	−	−	−	−	+	+	5
Zucker et al., 2017 [45]	+	+	+	+	+	−	−	−	+	+	+	7
Karatay et al., 2018 [46]	+	+	+	+	+	−	+	+	−	+	−	7
Mist et al., 2018 [47]	+	+	−	+	−	−	+	+	+	+	+	7
Castro-Sánchez et al., 2019 [6]	+	+	+	+	−	−	+	+	+	+	+	8
Yuksel et al., 2019 [48]	+	+	+	+	−	−	−	+	+	+	+	7
Ozen et al., 2019 [49]	+	+	+	+	−	+	−	+	+	+	+	8
Castro-Sánchez et al., 2020 [50]	+	+	+	+	+	−	+	+	+	+	+	9
Di Carlo et al., 2020 [51]	+	−	−	+	−	+	+	+	+	+	+	7
Garrido et al., 2020 [52]	+	+	+	−	−	+	+	+	+	+	+	8
Schweiger et al., 2020 [53]	+	+	+	+	−	−	+	+	+	+	+	8
Garrido-Ardila et al., 2021 [54]	+	+	+	+	−	−	+	+	+	+	+	8

1: Eligibility criteria were specified; 2: Subjects were randomly allocated to groups; 3: Allocation was concealed; 4: The groups were similar at baseline regarding the most important prognostic indicators; 5: There was blinding of all subjects; 6: There was blinding of all therapists who administered the therapy; 7: There was blinding of all assessors who measured at least one key outcome; 8: Measures of at least one key outcome were obtained from more than 85% of the subjects initially allocated to groups; 9: All subjects for whom outcome measures were available received the treatment or control condition as allocated or, where this was not the case, data for at least one key outcome was analyzed by “intention to treat”; 10: The results of between-group statistical comparisons are reported for at least one key outcome; 11: The study provides both point measures and measures of variability for at least one key outcome. +: Yes; −: No.

**Table 2:** Data Synthesis of the Studies applying Dry Needling in Patients with Fibromyalgia.

Study	Study Design	Sample Characteristics	Objectives	Interventions	Outcomes Assessed	Results	Conclusions
Casanueva et al., 2014 [36]	Randomized controlled trial	*n* = 110 patients with FM 110 women/10 men	To evaluate the short-term efficacy of dry needling therapy in patients severely affected by fibromyalgia	EG: Tender points dry needling once a week for 6 weeks + their medical treatment: (*n* = 60) 54 women and 6 men CG: Medical treatment: (*n* = 60) 56 women and 4 men	McGill Pain Questionnaire VAS of Pain PPT Myalgic score VAS of fatigue Global Subjective Improvement SF-36 Questionnaire	At the end of treatment, the EG showed significant differences in VAS of pain (*p* = 0.002), VAS of fatigue (*p* = 0.02), SF-36 (*p* = 0.0007), myalgic score (*p* = 0.0005), PPT (*p* = 0.002) and global subjective improvement (*p* = 0.00001). Six weeks after the end of the treatment, the dry needling group still showed significant differences in VAS of pain (*p* = 0.01), VAS of fatigue (*p* = 0.02), SF-36 (*p* = 0.01), myalgic score (*p* = 0.00001), PPT (*p* = 0.0004) and global subjective improvement (*p* = 0.00001).	Patients severely affected by fibromyalgia can obtain short-term improvements following weekly dry needling for 6 weeks.
Castro-Sánchez et al., 2017 [42]	Single-blind randomized controlled trial	*n* = 64 patients aged 27–58 years old with FM 59 women/5 men	To compare the effectiveness of dry needling versus cross tape on spinal mobility and MTrP in spinal muscles in patients with FM.	EG: Spinal muscles MTrPs Dry needling (*n* = 32) CG: Cross Tape application (*n* = 32) 4 interventions a week for 5 weeks	Spinal mobility MTrPs PPT	Significant differences between groups were achieved for the MTrPs in latissimus dorsi muscle (right axillary portion: F = 9.80, *p* = 0.003); multifidus muscle (right L2 level: F = 11.80, *p* = 0.001); quadratus lumborum (right lateral superficial upper: F = 6.67, *p* = 0.012; and right lateral superficial lower: F = 5.38, *p* = 0.024). In addition, significant differences between groups for the segmental amplitude thoracic spine in the standing erect position (F = 7.33, *p* = 0.009), and segmental amplitude of lumbar spine (F = 11.60, *p* = 0.001) in the sitting erect position.	Dry needling therapy reduces MTrP algometry on thoracic and lumbar muscles. Dry needling and cross tape approaches reported a similar effect size for spinal mobility measures in patients with FM
Castro-Sánchez et al., 2019 [6]	Single-blind randomized controlled trial	*n* = 64 patients aged 27–58 years old with FM 58 women/6 men	To compare the effectiveness of dry needling versus myofascial release on myofascial trigger points pain in cervical muscles, quality of life, impact of symptoms pain, quality of sleep, anxiety, depression and fatigue in patients with FM	EG: Cervical muscles MTrPs Dry needling (*n* = 32) CG: Myofascial release (*n* = 32) Baseline and post treatment after four weeks of interventions	PPT of MTrP in cervical muscles Quality of life Impact of fibromyalgia Quality of sleep Intensity of pain Anxiety Depression Fatigue	Significant improvement was found in most pain pressure thresholds of the myofascial trigger points in cervical muscles in the dry needling group compared to myofascial release (*p* < 0.05). Similarly, these differences between groups were found for the components of quality of life of physical function (F = 12.74, *p* = 0.001), physical role (F = 11.24, *p* = 0.001), body pain (F = 30.26, *p* < 0.001), general health (F = 15.83, *p* < 0.001), vitality (F = 13.51, *p* = 0.001), social function (F = 4.73, *p* = 0.034), emotional role (F = 8.01, *p* = 0.006) and mental health (F = 4.95, *p* = 0.030). Similar results were achieved for total impact of FM symptoms (F = 42.91, *p* < 0.001), quality of sleep (F = 11.96, *p* = 0.001), state anxiety (F = 7.40, *p* = 0.009), and trait anxiety (F = -14.63, *p* < 0.001), hospital anxiety and depression (F = 20.60, *p* < 0.001), general pain intensity (F = 29.59, *p* < 0.001) and fatigue (F = -25.73, *p* < 0.001).	Dry needling therapy showed higher improvements in comparison with myofascial release therapy for pain pressure thresholds, the components of quality of life of physical role, body pain, vitality and social function, as well as the total impact of FM symptoms, quality of sleep, state and trait anxiety, hospital anxiety-depression, general pain intensity and fatigue. Implications for rehabilitation: Dry needling therapy reduces myofascial trigger point pain in the short term in patients with fibromyalgia syndrome. This therapeutic approach improves anxiety, depression, fatigue symptoms, quality of life and sleep after treatment. Dry needling and myofascial release therapies decrease intensity of pain, and the impact of fibromyalgia symptoms in this population.
Castro-Sánchez et al., 2020 [50]	Randomized controlled trial	*n* = 74 patients with FM 60 women/14 men	To compare the effects of dry needling and transcutaneous electrical nerve stimulation (TENS) on pain intensity, heart rate variability, galvanic response and oxygen saturation (SpO_2_)	EG: Dry needling (*n* = 37) CG: TENS (*n* = 37) Patients evaluated at baseline and after 6 weeks of treatment.	Pain intensity Heart rate variability Galvanic skin response Oxygen saturation Photoplethysmography	Significant differences between groups were found for the sensory dimension of pain, affective dimension of pain, total dimension of pain, visual analogue scale (VAS) and present pain intensity (PPI) (*p* = 0.001). In addition, significant differences between groups were achieved for very low frequency power of heart rate variability (*p* = 0.008) and low frequency power (*p* = 0.033). There were no significant differences in dry needling versus TENS groups on the spectral analysis of the photoplethysmography and oxygen saturation.	Dry needling therapy and TENS reduced pain attributable to MTrPs in patients with fibromyalgia, with greater improvements reported in the dry needling group across all dimensions of pain. Additionally, there were between-intervention differences for several parameters of heart rate variability and galvanic skin responses

EG: Experimental Group; CG: Control Group; PPT: Pressure Pain Thresholds; TENS: Transcutaneous Electrical Nerve Stimulation; VAS: Visual Analogue Scale.

**Table 3:** Data Synthesis of the Studies applying Acupuncture in Patients with Fibromyalgia.

Study	Study Design	Sample Characteristics	Objectives	Interventions	Outcomes Assessed	Results	Conclusions
Collazo-Chao et al., 2010 [32]	Quasi-experimental study	*n* = 42 female patients with fibromyalgia	To evaluate the effectiveness of acupuncture as a combined modality therapy in reducing pain in paired groups (before-after) of patients with fibromyalgia in conditions of clinical practice and to determine the extent to which analgesic consumption is reduced in these patients after a cycle of acupuncture and to identify the adverse reactions due to acupuncture	EG: Acupuncture application (*n* = 42) 10 sessions	Pain intensity Pain frequency Analgesic intake Disability Sleep quality	After application of an acupuncture cycle, the total pain scale score decreased by 51.5%, intensity by 46.9%, frequency by 41.3%, analgesic intake by 54.3% and disability by 46.2%; sleep improved by 64.4%. Drug intolerance was found in 26.2% of the patients.	Acupuncture is highly effective in the combined treatment of fibromyalgia and consumes scant resources. The reduction in drug intake in patients with fibromyalgia decreases adverse effects and healthcare costs and improves quality of life and user satisfaction.
Collazo-Chao et al., 2012 [33]	Randomized controlled trial	*n* = 120 female patients with fibromyalgia	To evaluate the effectiveness of acupuncture and traditional Chinese dietary therapy as single therapies in reducing pain and improving quality of life in patients with fibromyalgia versus combined treatment with acupuncture and traditional Chinese dietary therapy	EG1: Acupuncture and dietary therapy (*n* = 30) EG2: Acupuncture (*n* = 30) EG3: Dietary therapy (*n* = 30) CG (*n* = 30) 10 sessions for 6 months follow-up	VAS of pain FIQ	Treatment with traditional Chinese dietary therapy alone did not improve fibromyalgia symptoms. The combination of traditional Chinese dietary therapy with acupuncture produced significant improvements (*p* < 0.05). At 6 months, the best results were achieved in the acupuncture group.	The application of TCDT together with acupuncture is an effective measure to improve the therapeutic approach, although acupuncture produced the greatest improvements.
Iannuccelli et al., 2012 [34]	Quasi-experimental study	*n* = 30 female patients with fibromyalgia	To combine two different acupunctural methods (the somatic and abdominal one) in the treatment of 30 consecutive female patients with fibromyalgia and to evaluate the reduction in pain and the improvement in well-being state	EG1: Somatic acupuncture (*n* = 15) EG2: Abdominal acupuncture (*n* = 15) Weekly sessions for 10 weeks	Pain, disability, psychologic health and quality of life by using: VAS of pain FIQ FAS HAQ ZSAS ZSDS	The results showed a statistically significant reduction in the number of tender points and in pain. Moreover, we observed a statistically significant reduction in FIQ, FAS, HAQ, disease activity VAS, ZSAS, ZSDS at the end of the treatment.	The combination of two types of acupuncture could be a useful complementary treatment in FM patients, not only to control pain but also to improve associated symptoms and quality of life. As a result, acupuncture could be very useful to relieve pain in a multidisciplinary setting.
Collazo-Chao et al., 2013 [35]	Randomized controlled trial	*n* = 62 female patients with fibromyalgia	To assess and compare the effectiveness of acupuncture and scalp acupuncture as exclusive therapies for alleviating pain and improving the quality of life in patients with fibromyalgia	EG1: Acupuncture (*n* = 31) EG2: Scalp acupuncture (*n* = 31) 10 sessions for 6 months follow-up	VAS of pain FIQ	The scalp acupuncture group showed significant differences on all variables compared to the acupuncture one, except for sleeping problems.	The scalp acupuncture protocol showed a remarkably higher effectiveness than the acupuncture protocol for treating fibromyalgia.
Collazo-Chao et al., 2014 [37]	Randomized controlled trial	*n* = 99 female patients with fibromyalgia	To assess the effectiveness of a moxibustion protocol and compare it to that of acupuncture and scalp acupuncture regarding alleviation of pain and improvement in the standard of living in patients with fibromyalgia	EG1: Acupuncture (*n* = 33) EG2: Moxibustion (*n* = 33) EG3: Scalp acupuncture (*n* = 33) 10 sessions for 6 months follow-up	VAS of pain FIQ	The scalp acupuncture group showed significant differences in all variables compared to the other two groups after 6 months; particularly, the pain scale values decreased by 28.23% and those of the VAS by 20%. No differences were found on the FIQ in any of the groups. There were significant differences (*p* < 0.01) after 6 months in analgesic use, favoring moxibustion over acupuncture.	The scalp acupuncture protocol used showed notably more effectiveness than the moxibustion protocol and acupuncture treatment after syndromic diagnosis in the management of fibromyalgia.
Stival et al., 2014 [38]	Double-blinded randomized controlled trial	*n* = 36 patients with fibromyalgia 31 women/5 men	To evaluate the efficacy of acupuncture in the treatment of fibromyalgia, considering the immediate response of the VAS of pain as its primary outcome	EG: Acupuncture (*n* = 21) PG: Sham acupuncture (*n* = 15)	VAS of pain	The variation between the final and initial VAS values was −4.36 ± 3.23 (*p* = 0.0001) in the treatment group and −1.70 ± 1.55 in the control group (*p* = 0.06). The difference in terms of amplitude of variation of VAS (initial—final VAS) among groups favored the actual procedure (*p* = 0.005). The effect size for the treatment group was d = 1.7, which is considered a large effect. Although small, the statistical power of the sample for these results was very relevant (94.8%).	Acupuncture has proven effective in the immediate pain reduction in patients with fibromyalgia, with a quite significant effect size.
Weber et al., 2015 [39]	Randomized controlled trial	*n* = 120 female patients with fibromyalgia	To investigate the effect of music combined with vibration on acupuncture points for the treatment of fibromyalgia	EG1: Bach’s music sequence EG2: Vibratory stimuli on a combination of acupuncture points on the skin EG3: Simultaneous and synchronized vibratory stimuli and Bach’s sequence CG: No stimulation 5 sessions on alternate days	FIQ HAQ	All groups showed a significant improvement in FIQ and HAQ scores at the evaluation after the intervention. The complete group exhibited the best result on both the FIQ and HAQ (*p* < 0.001), and the improvement in HAQ score was significant (*p* < 0.004).	The placebo effect in FM may be substantial. However, comparison between groups revealed that the complete group had the greatest reduction in both FIQ and HAQ, with a significant improvement in HAQ, suggesting that the combined use of music and vibration exerts a greater effect on FM symptoms.
Dias et al., 2016 [40]	Non-randomized controlled trial	*n* = 30 female patients with fibromyalgia	To compare three classical traditional Chinese medicine therapies: acupuncture, electroacupuncture and moxibustion in the management of pain and promotion of quality of life in patients with fibromyalgia	EG1: Acupuncture (*n* = 10) EG2: Electroacupuncture (*n* = 10) EG3: Moxibustion (*n* = 10) One session a week for 8 weeks	Pain: PPT WBFPS Quality of life: SF-36	There was no significant improvement in pain or reduction in tender points in any of the groups studied, at the end of the eighth session. Significant improvement in quality of life was perceived in vitality (after acupuncture treatment) and in mental health (after electroacupuncture and moxibustion treatments).	Traditional Chinese medicine therapies promoted an improvement in the quality of life in two areas (vitality and mental health) in women with fibromyalgia.
Vas et al., 2016 [41]	Double-blinded randomized controlled trial	*n* = 153 female patients with fibromyalgia	To evaluate the efficacy of an individualized acupuncture protocol for patients with fibromyalgia	EG: Acupuncture (*n* = 73) SG: Sham acupuncture (*n* = 80) Interventions once a week for 10 weeks	VAS of pain FIQ SF-12 questionnaire HDRS	Intention-to-treat analysis revealed that the decrease in pain intensity at 10 weeks was greater (*p* = 0.001) in the EG group (−41.0%, 95% CI −47.2% to −34.8%) than in the SG group (−27.1%, 95% CI −33.2% to −20.9%). During the follow-up period (6 and 12 months), significant differences (*p* < 0.01) in favor of the EG group persisted at 12 months (EG: −19.9%, 95% CI −24.6% to −15.1%; vs. SG: −6.2%, 95% CI −11.2% to −1.2%).	Individualized acupuncture treatment in primary care in patients with fibromyalgia proved efficacious in terms of pain relief, compared with placebo treatment. The effect persisted at 1 year, and its side effects were mild and infrequent. Therefore, the use of individualized acupuncture in patients with fibromyalgia is recommended.
Iannuccelli et al., 2017 [43]	Quasi-experimental study	*n* = 30 patients with fibromyalgia and 20 healthy subjects to compare baseline scores	To assess the effects of an acupuncture cycle on serum NPY levels in patients with FM and identify possible correlations between its serum levels and clinical and clinimetric parameters	Acupuncture (*n* = 30)	Serum NPY levels NTP VAS of pain	The baseline serum NPY levels of the patients were higher than those of the controls. They had significantly increased by the end of the treatment, when there was also a statistically significant reduction in pain, the number of tender points numbers and the clinimetric scores.	These findings confirm the analgesic properties of acupuncture as a complementary treatment in FM and indicate that NPY could play a role in pain modulation.
Ugurlu et al., 2017 [44]	Randomized controlled trial	*n* = 50 female patients with fibromyalgia	To determine and to compare the efficacy of real acupuncture with sham acupuncture on fibromyalgia treatment	EG: Acupuncture (*n* = 25) SG: Sham acupuncture (*n* = 25) Interventions were 3 sessions in the first week, twice a week for 2 weeks and once a week in the following 5 weeks (total: 12 sessions)	VAS of pain SF-36 questionnaire FIQ BDI FSS	Both groups improved significantly in all parameters 1 month after the first session and this improvement persisted 2 months after the first session (*p* < 0.05). However, real acupuncture group had better scores than sham acupuncture score in terms of all VAS scores, BDI and FIQ scores either 1 or 2 months after the first session (all *p* < 0.05).	Acupuncture significantly improved pain and symptoms of fibromyalgia. Although sham effect was important, real acupuncture treatment seems to be effective in treatment of fibromyalgia.
Zucker et al., 2017 [45]	Randomized controlled trial	*n* = 114 patients with fibromyalgia	To assess the treatment response to verum and sham acupuncture on pressure pain tenderness in fibromyalgia patients	EG: Acupuncture (*n* = 59) SG: Sham acupuncture (*n* = 55) Interventions were from once a week to three times a week, increasing in three-week blocks for a total of 18 treatments	VAS of pain Needle sensation	Participants who had higher pain pressure thresholds had greater reduction in clinical pain following verum acupuncture, while participants who had lower pain pressure thresholds showed better analgesic response to sham acupuncture. Moreover, patients with lower pressure pain thresholds had exacerbated clinical pain following verum acupuncture. Similar relationships were observed for sensitivity to acupuncture needling.	Acupuncture efficacy in fibromyalgia may be underestimated, and a more personalized treatment for fibromyalgia may also be possible.
Karatay et al., 2018 [46]	Randomized controlled trial	*n* = 75 female patients with fibromyalgia	To evaluate the effects of acupuncture treatment on serum levels of serotonin and substance *p* as well as on clinical parameters in patients with fibromyalgia	EG: Acupuncture (*n* = 25) SG1: Sham acupuncture (*n* = 25) SG2: Simulated acupuncture (*n* = 25) Interventions were semiweekly for 4 weeks	NTP VAS FIQ BDI NHP	Serum serotonin values increased significantly after treatment in EG and SG1 (*p* < 0.001 and *p* < 0.01, respectively). The increase in the EG was also different from both of the other groups (*p* < 0.01). While substance *p* levels decreased in the EG, they increased in the SG2 (*p* = 0.001). In the EG, significant improvements were found in almost all clinical outcomes after treatment. These usually continued for three months. In the SG1, there were also significant changes in the NTP, VAS, FIQ and BDI scores after treatment. Improvements in the NTP and FIQ scores lasted for three months. In the SG, significant improvements were found only in the NTP, VAS and BDI scores after treatment.	Acupuncture, rather than sham or placebo acupuncture, may lead to long-term improvements in clinical outcomes and pain neuromediator values. Changes in serum serotonin and substance P levels may be a valuable explanation for acupuncture mechanisms in fibromyalgia treatment.
Mist et al., 2018 [47]	Randomized controlled trial	*n* = 30 female patients with fibromyalgia	To test the treatment effect of group acupuncture vs. group education in persons with fibromyalgia	EG1: Acupuncture (*n* = 16) EG2: Group education (*n* = 14) Twice a week for 10 weeks	Revised FIQ GFI	FIQR total, FIQR pain and Global Fatigue Index all had clinically and statistically significant improvement in the group receiving acupuncture at end of treatment and four weeks post-treatment but not in participants receiving group education between groups.	Compared with education, group acupuncture improved global symptom impact, pain and fatigue. Furthermore, it was a safe and well-tolerated treatment option, improving a broader proportion of patients than current pharmaceutical options.
Yuksel et al., 2019 [48]	Randomized controlled trial	*n* = 42 patients with fibromyalgia	To evaluate the effects of acupuncture and TENS applications on the quantitative EEG changes and to evaluate their therapeutic effects in patients with fibromyalgia	EG1: Acupuncture (*n* = 21) EG2: TENS (*n* = 21)	VAS of pain BDI FIQ Tender points PPT EEG	In the TENS group, after the treatment, an increase was observed in the alpha power of the left anterior region as well as a decrease in pain scores. In the acupuncture group, an increase was determined in the alpha power of the right and left posterior regions as well as a decrease in pain score after the treatment. The power of low- and moderate-frequency waves on resting EEG was decreased in the patients with fibromyalgia. Decreased pain and increased inhibitor activity were found on EEG after TENS and acupuncture applications.	TENS and acupuncture applications seem to be beneficial in fibromyalgia patients.
Ozen et al., 2019 [49]	An experimental effectiveness comparative study	*n* = 44 female patients with fibromyalgia	To compare the effects of physiotherapy modalities with those of acupuncture on pain, daily function and quality of life in fibromyalgia patients.	EG1: TENS (*n* = 22) 15 sessions Five consecutive sessions per week EG2: Acupuncture (*n* = 22) 10 sessions Once every 2 days for 3 weeks	SF-MPQ VAS of pain FIQ	There was a reduction in all SF-MPQ domains and FIQ scores after treatment in both the physical therapy and acupuncture groups. There was no difference in pre- and post-treatment scores between the two groups.	Physical therapy modalities and acupuncture can be effectively used in the treatment of fibromyalgia. Even though one treatment option was not found to be more beneficial than the other, longer post-treatment follow-up may help determine the superior treatment option.
Di Carlo et al., 2020 [51]	Quasi-experimental study	*n* = 96 patients with fibromyalgia 85 women/11 men	To explore the role of acupuncture, in terms of efficacy on main disease severity measures and pain features, in patients with nonresponsive disease despite optimal drug therapy	EG: Acupuncture (*n* = 96) Once a week during 8 weeks	FIQ PCS PDQ PHQ	At the end of the eight-week treatment, patients experienced a significant improvement in all evaluated parameters (for FIQ, PDQ and PHQ *p* < 0.0001; for PCS *p* = 0.001).	It can be stated that acupuncture can be proposed also in phases of high severity of disease. Intervention with multimodal strategies, including acupuncture, could be of great benefit to patients.
Garrido et al., 2020 [52]	Randomized controlled trial	*n* = 135 female patients with fibromyalgia	To investigate the effectiveness of a core stability training physiotherapy program vs. acupuncture for the management of balance and functional capacity impairments of women with Fibromyalgia	EG1: Core stability physiotherapy program (*n* = 45) EG2: Acupuncture (*n* = 45) CG (*n* = 45) 13 weeks duration	Static Balance Berg Balance Scale Timed up and go test 10 min walk test FHAQ FIQ	The results showed statistically significant improvements in the acupuncture and physiotherapy groups vs. the control group at week 6 regarding Berg Balance Scale (*p* = 0.00, both groups), timed up and go test (*p* = 0.00 and *p* = 0.01, respectively) and 10 m walk test at comfortable speed (*p* = 0.02 and *p* = 0.03, respectively). The 10 m walk test at maximum speed showed significance when comparing the physiotherapy and control group (*p* = 0.03). However, no significant differences were found between the physiotherapy and the acupuncture groups. In relation to functional capacity, the improvements achieved after the treatments were not statistically significant.	Core stability-based physiotherapy and acupuncture improve dynamic balance and postural control in women with fibromyalgia.
Schweiger et al., 2020 [53]	Randomized controlled trial	*n* = 54 female patients with fibromyalgia	To compare two alternative treatments (nutraceutical and acupuncture) in fibromyalgia patients through a randomized clinical trial.	EG1: Nutritional combination containing coenzyme Q10, vitamin D, Alpha-lipoic acid, magnesium, and tryptophan (*n* = 21) EG2: Acupuncture (*n* = 34) 3 months duration	FIQ FSS	EG1 showed a statistically significant reduction in pain 1 month after the start of therapy (T1, *p* = 0.025), strengthened after 3 months with maintenance of treatment (*p* = 0.012). The efficacy in reducing pain was apparent in the Acupuncture Group at all post-treatment determinations and at follow-up (T1 and T2 *p* ≤ 0.001). Regarding quality of life, improvement in FIQ-R and FSS values was revealed in both groups.	The nutritional combination assessed seems to be an effective option to for patients with fibromyalgia. Our experience confirmed also the validity of acupuncture in these patients. Considering the complexity of the management of fibromyalgia patients, our results suggest a cyclical and sequential, or even concurrent, treatment with different approaches to improve the efficacy and the compliance of patients to long-term treatment.
Garrido-Ardila et al., 2021 [54]	Randomized controlled trial	*n* = 135 female patients with fibromyalgia	To assess the effectiveness of a core stability training physiotherapy program compared to an acupuncture treatment on quality of life, pain, joint stiffness, difficulty to work and depression of women with fibromyalgia	EG1: Core stability physiotherapy program (*n* = 45) EG2: Acupuncture treatment group CG: No treatment 13 weeks of duration	FIQ Pain intensity Joint Stiffness Difficulty to work Depression	Only the difficulty to work measure in the acupuncture group showed a slight decrease at week 13. In particular, mean (±SD) Spanish Fibromyalgia Impact Questionnaire score at 6 weeks was 62.89 ± 16.91 for the physiotherapy group, 62.5 ± 18.09 for the acupuncture group and 67.45 ± 17.07 for the control group. However, these improvements were not statistically significant.	Core-stability-based physiotherapy and acupuncture showed non-significant improvements in quality of life, pain, joint stiffness, difficulty to work and depression in women with fibromyalgia.

**Table 4:** GRADE Evidence.

Number of Studies	Risk of Bias	Inconsistency	Indirectness of Evidence	Imprecision	Publication Bias	Quality of Evidence	MD [95% CI] or SMD [95% CI]
Pain intensity (Short-term follow-up)							
Overall effect (*n* = 15, *N* = 1033)	No	Very Serious (I^2^ = 87%)	No	No	No	Low	MD: −1.18 [−1.91, −0.45] * SMD: −0.55 [−0.89, −0.21] *
Acupuncture (*n* = 11, *N* = 711)	No	Very Serious (I^2^ = 84%)	No	No	No	Low	MD: −0.70 [−1.54, 0.14] SMD: −0.30 [−0.65, 0.06]
Dry Needling (*n* = 4, *N* = 322)	No	Serious (I^2^ = 78%)	No	No	No	Moderate	MD: −2.36 [−3.27, −1.46] * SMD: −1.20 [−1.66, −0.75] *
Pain intensity (Mid-term follow-up)							
Acupuncture (*n* = 3, *N* = 174)	No	Serious (I^2^ = 72%)	No	Serious	No	Moderate	MD: −0.56 [−1.72, 0.61] SMD: −0.26 [−0.82, 0.29]
Pain intensity (Long-term follow-up)							
Acupuncture (*n* = 5, *N* = 423)	No	Very Serious (I^2^ = 81%)	No	No	No	Low	MD: −0.86 [−1.78, 0.07] SMD: −0.42 [−0.88, 0.04]
Fibromyalgia Impact Questionnaire (Short-term follow-up)							
Overall effect (*n* = 11, *N* = 817)	No	Very Serious (I^2^ = 90%)	No	No	No	Low	MD: −6.19 [−13.67, 1.29] SMD: −0.36 [−0.77, 0.05]
Acupuncture (*n* = 9, *N* = 633)	No	Very Serious (I^2^ = 91%)	No	No	No	Low	MD: −3.96 [−12.60, 4.68] SMD: −0.26 [−0.75, 0.22]
Dry Needling (*n* = 2, *N* = 184)	No	Serious (I^2^ = 73%)	No	Serious	No	Low	MD: −16.03 [−28.20, −3.86] * SMD: −0.75 [−1.22, −0.28] *
Fibromyalgia Impact Questionnaire (Mid-term follow-up)							
Acupuncture (*n* = 2, *N* = 107)	No	Serious (I^2^ = 81%)	No	Serious	No	Low	MD: −4.08 [−18.08, 9.93] SMD: −0.25 [−1.11, 0.61]
Fibromyalgia Impact Questionnaire (Long-term follow-up)							
Acupuncture (*n* = 5, *N* = 423)	No	Very Serious (I^2^ = 80%)	No	No	No	Low	MD: −7.57 [−14.92, −0.22] * SMD: −0.44 [−0.85, −0.02] *
Sleep (Short-term follow-up)							
Overall effect (*n* = 6, *N* = 534)	No	Very Serious (I^2^ = 80%)	No	No	No	Low	SMD: −0.38 [−0.78, 0.02]
Acupuncture (*n* = 4, *N* = 350)	No	Very Serious (I^2^ = 86%)	No	No	No	Low	SMD: −0.31 [−0.93, 0.30]
Dry Needling (*n* = 2, *N* = 184)	No	No (I^2^ = 0%)	No	Serious	No	Moderate	SMD: −0.51 [−0.81, −0.22] *
Sleep (Mid-term follow-up)							
Acupuncture (*n* = 2, *N* = 107)	No	Very Serious (I^2^ = 86%)	No	Very serious	No	Very Low	SMD: −0.25 [−1.30, 0.79]
Sleep (Long-term follow-up)							
Acupuncture (*n* = 2, *N* = 354)	No	Very Serious (I^2^ = 81%)	No	No	No	Low	SMD: −0.31 [−0.82, 0.21]
Depression (Short-term follow-up)							
Overall effect (*n* = 6, *N* = 512)	No	Serious (I^2^ = 76%)	No	No	No	Moderate	SMD: −0.52 [−0.90, 0.14] *
Acupuncture (*n* = 4, *N* = 328)	No	Very Serious (I^2^ = 85%)	No	No	No	Low	SMD: −0.68 [−1.31, −0.06] *
Dry Needling (*n* = 2, *N* = 184)	No	No (I^2^ = 0%)	No	Serious	No	Moderate	SMD: −0.29 [−0.58, 0.00]
Pressure pain threshold (Short-term follow-up)							
Overall effect (*n* = 4, *N* = 406)	No	No (I^2^ = 0%)	No	No	No	High	SMD: 0.81 [0.60, 1.01] *
Acupuncture (*n* = 1, *N* = 162)	No	No	No	No	No	Very Low	SMD: 0.68 [0.36, 1.00] *
Dry Needling (*n* = 3, *N* = 244)	No	No (I^2^ = 0%)	No	Serious	No	Moderate	SMD: 0.89 [0.63, 1.16] *

Risk of bias: No: Most information is from results at low risk of bias; Serious: Crucial limitation for one criterion, or some limitations for multiple criteria, sufficient to lower confidence in the estimate of effect; Very Serious: Crucial limitation for one or more criteria sufficient to substantially lower confidence in the estimate of effect. Inconsistency: Serious: I^2^ > 40%; Very Serious: I^2^ > 80%. Indirectness of Evidence: No indirectness of evidence was found in any study. Imprecision (based on sample size): Serious: *n* < 250 subjects; Very Serious: *n* < 250 and the estimated effect is little or absent. Publication bias (based on funnel plots): No publication bias was observed based on funnel plots (not shown because the lower number of studies < 10). * *p* < 0.05

## Data Availability

All data derived from this study are presented in the text.
